# Blind and Secured Adaptive Digital Image Watermarking Approach for High Imperceptibility and Robustness

**DOI:** 10.3390/e23121650

**Published:** 2021-12-08

**Authors:** Priyanka Singh, Kilari Jyothsna Devi, Hiren Kumar Thakkar, José Santamaría

**Affiliations:** 1 Department of Computer Science and Engineering, SRM University, Amaravati 522508, India; priyanka.s@srmap.edu.in (P.S.); jyothsna_devi@srmap.edu.in (K.J.D.); 2 Department of Computer Engineering, Marwadi University, Rajkot 360006, India; 3 Department of Computer Science, University of Jaén, 23071 Jaén, Spain

**Keywords:** IWT-SVD, digital image watermarking, adaptive embedding, adaptive scaling factor, pseudo random key, firefly, GA, ABC

## Abstract

In the past decade, rapid development in digital communication has led to prevalent use of digital images. More importantly, confidentiality issues have also come up recently due to the increase in digital image transmission across the Internet. Therefore, it is necessary to provide high imperceptibility and security to digitally transmitted images. In this paper, a novel blind digital image watermarking scheme is introduced tackling secured transmission of digital images, which provides a higher quality regarding both imperceptibility and robustness parameters. A block based hybrid IWT- SVD transform is implemented for robust transmission of digital images. To ensure high watermark security, the watermark is encrypted using a Pseudo random key which is generated adaptively from cover and watermark images. An encrypted watermark is embedded in randomly selected low entropy blocks to increase the security as well as imperceptibility. Embedding positions within the block are identified adaptively using a Blum–Blum–Shub Pseudo random generator. To ensure higher visual quality, Initial Scaling Factor (ISF) is chosen adaptively from a cover image using image range characteristics. ISF can be optimized using Nature Inspired Optimization (NIO) techniques for higher imperceptibility and robustness. Specifically, the ISF parameter is optimized by using three well-known and novel NIO-based algorithms such as Genetic Algorithms (GA), Artificial Bee Colony (ABC), and Firefly Optimization algorithm. Experiments were conducted for the proposed scheme in terms of imperceptibility, robustness, security, embedding rate, and computational time. Experimental results support higher effectiveness of the proposed scheme. Furthermore, performance comparison has been done with some of the existing state-of-the-art schemes which substantiates the improved performance of the proposed scheme.

## 1. Introduction

Due to the pioneering of 5G technology in telecommunication, use of multimedia content, such as audio, images, and video, has increased many fold. Transmission of multimedia content through the Internet on public domains such as social networks, e-health, e-commerce and e-business applications have several barriers [1]. Digital images are more popular in carrying information through the Internet [2]. The immoderate usage of internet duplication, unauthorized access, and tampering of digital images has increased excessively. Therefore, it has become necessary to maintain the authenticity, confidentiality, and integrity of digital images. One of the most feasible solutions to safeguard the digital images is Digital Image Watermarking (DIW) [3]. DIW is the process of embedding a watermark in the form of text, image, or binary data in a cover image to produce a watermarked image. The watermark is embedded in a spatial or spectral domain. However, it is observed that spectral domain watermark embedding is more robust than the spatial domain watermarking [4,5]. Robust embedding can sustain malicious signal processing attacks, and they are more suitable for secured transmission [6]. In an efficient watermarking scheme, characteristics such as imperceptibility, robustness, and embedding rate play an instrumental role [7]. In particular, imperceptibility is defined as the visual quality of cover and watermarked images, and, ideally, both should be the same. A DIW scheme is considered robust, and provided original and extracted watermarks are almost similar even under attacks. Embedding rate is the ratio between the area of watermark (in bits) and the area of the cover image (in pixels) [8]. However, it is challenging to satisfy these characteristics at the same time and they are always a trade-off. In the last few years, researchers have suggested various schemes to balance this trade-off. One of the prominent solutions is to choose a strong scaling factor (α) for embedding the watermark. Nature Inspired Optimization (NIO) algorithms have become a very promising scheme to address this issue. Specifically, an effective fitness function can assist in generating a strong scaling factor [9]. However, it is still a challenge to achieve an optimal design for the objective function due to the inherent complex nature of the problem being involved. Watermark security is another important characteristic of watermarking. It is important to protect secret data during its transmission through the internet. To ensure the security of the information, the majority of the researchers have proposed Principle Component (PC) or Singular Value (SV) insertion, the use of hashing techniques, and encryption approaches, but they are less secure approaches. Nowadays, encryption is the most popular security approach [10]. Chaotic maps are often used for encryption, but it has limitations of hyper tuning issues. Therefore, there is a need to develop a strong encryption approach. To address the issues discussed above, a novel blind adaptive DIW scheme is proposed for secured watermark transmission with higher imperceptibility, robustness, and an embedding rate with an optimum computational cost.

The structure of the paper is as follows: Section 2 is devoted to reviewing the State-of-The-ART (SoTA) in the field. Next, a detailed description of the proposed scheme is introduced in Section 3. Section 4 provides a broad discussion on the results reported in the experimental section. Finally, some of those more relevant results and future investigations are accordingly summarized in Section 5.

## 2. Brief Overview

Existing DIW schemes mainly focus on achieving higher imperceptibility, robustness, and embedding rate by embedding the watermark in spatial and spectral domains. The DIW schemes proposed in [11,12,13,14] utilize spatial domain techniques. In general, spatial domain techniques are imperceptible but less robust. Therefore, researchers have also explored hybrid transform watermarking schemes for high imperceptibility and robustness, such as DWT-SVD [15,16,17,18], DWT-DCT [19,20], RDWT-SVD [21], and IWT-SVD [22,23]. The schemes proposed in [22,23] have high imperceptibility but lag behind in robustness, which can be attributed to the trade-off between watermarking characteristics. To balance this trade-off, the scaling factor for watermark embedding and extraction is suggested [11,12,13,16,18,20,23,24,25]. However, determining the scaling factor is challenging. In addition to that, using a constant scaling factor may not be effective for all images. Some researchers have offered scaling factor optimization strategies to overcome these problems. Schemes proposed in [15,17,21,22,26,27,28] use NIO algorithms such as MACO, GA, Firefly, ABC, GDPSO and ACO, respectively, for scaling factor optimization. Scaling factor is optimized using QIM in [19] and the fuzzy logic system in [12]. However, the schemes in [22,26] use optimized scaling factor but are less robust. Adaptive embedding techniques to ensure high imperceptibility and robustness are also suggested [16,29]. Watermark security is very important for watermark applications such as IoMT, Telemedicine, IoT, Big Data, cloud computing, and blockchain technology. However, watermark security is either overlooked or less focused on in most of the DIW schemes. However, the schemes presented in [12,15,17,21,22,26] optimize watermarking characteristics but ignore watermark security. For watermark security, schemes in [16,27,28,29,30,31,32] use entropy, histogram, pseudo random key, DDFA, d-sequence, and GBA, respectively, for adaptively locating embedding blocks, but provide lower security. Similarly, schemes proposed in [11,14,18,20,30,31] have high imperceptibility but are less secure. However, the techniques presented in [24,28,33] achieve great imperceptibility, robustness, and security at the expense of a large computational cost. Another way of ensuring watermark security is to use cryptographic techniques. An Arnold map is used for watermark security in [19,28], although the Arnold map has a low iteration value. A chaotic map is used in schemes [13,23,34], but a chaotic map has an issue with the hyper tuning parameter.

**Motivation and contribution of the proposed scheme:** A study of related watermarking schemes reveals that most of the DIW schemes underestimate watermark security, whereas some have used cryptographic techniques like chaotic and Arnold map, which suffer from the iteration parameter and hyper tuning issues, respectively. In addition to that, the embedding positions in cover image are predetermined in most of the existing schemes, which further dilutes the confidentiality of watermark. In addition, most of the watermarking schemes use a constant scaling factor for embedding. Few schemes suggest optimization of the scaling factor using NIO algorithms to achieve high watermarking characteristics. Choosing a constant initial scaling factor for all image modalities may degrade watermarking characteristics. To address these challenges, a novel DIW scheme is proposed in this paper. The proposed scheme is motivated by Ansari and Pant [18], Moeinaddini [31], Singh and Bhatnagar [32], Sharma and Mir [27], and Zainol et al. [23]. A contribution of the proposed scheme is as follows:

1. High Watermark Security: The proposed scheme ensures twofold watermark security by encrypting the watermark and then embedding it in randomly selected positions in transformed cover image blocks. The watermark is partitioned into odd and even position pixel vectors. These vectors are encrypted by using pseudo random keys generated adaptively from the mean of IWT transformed sub-bands (LL, LH, HL, HH) of the cover image and the sum of the watermark image and key generation algorithm. The encrypted watermark is embedded in randomly selected pixel positions within the adaptively selected block using a Blum–Blum–Shum pseudo random generator.
2. High Imperceptibility: In the proposed scheme, an Initial scaling factor (ISF) is adaptively generated from the cover image using a fuzzy based texture range filter to ensure higher imperceptibility. In addition, adaptive selection of low entropy blocks for embedding, increasing the imperceptibility.
3. High Robustness: A hybrid IWT–SVD transformation is used in the proposed scheme to ensure high robustness. Adaptive ISF generation and block selection for embedding also improve the robustness
4. Scaling Factor Optimization: To improve imperceptibility, robustness and balancing the trade-off in watermarking characteristics, optimization of ISF is proposed, if the computational cost is not the major concern in the application. NIO algorithms such as GA, ABC, and FO can be used for optimization.

## 3. Proposed Work

The proposed scheme is comprised of the following three main modules: (i) Watermark Embedding and Extraction; (ii)Watermark Encryption and Decryption; and (iii) ISF generation and optimization. Each module is accordingly described in the next subsections.

### 3.1. Watermark Embedding and Extraction

**Figure 1:**
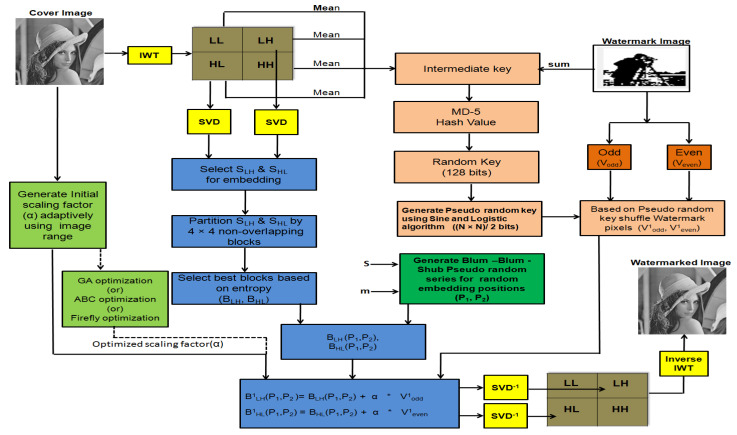
Block diagram for the Watermark Embedding process.

In the proposed scheme, IWT-SVD hybrid transform is applied on the cover image (C) of size M×N. A binary watermark (W) of size P×Q is encrypted and embedded randomly in the low entropy non-overlapping blocks of the cover image to achieve higher imperceptibility, robustness, and security. A block diagram of the proposed watermark embedding is shown in Figure 1. The steps for watermark embedding are provided in Algorithm 1 and explained as follows:

Watermark Embedding: Firstly, 1-IWT transform is applied on C to obtain LL, LH, HL and HH sub-bands. IWT is applied to achieve higher imperceptibility as well as higher robustness against compression algorithms and filtering. The LL sub-band contains approximate sub-images, whereas LH, HL and HH sub-bands have fringe information of the image. Therefore, LH and HL sub-bands are selected for watermark embedding. The selected LH and HL sub-bands are divided into 4×4 blocks, and block-wise entropy is calculated and stored in a vector V. Image entropy is the randomness measure, and it is used to characterize the texture of the image. The relation used for calculating the image entropy is as follows:

(1) $$Entropy=-\sum_{i=1}^{256}PB_{i}\;log_{2}\;PB_{i}$$

where PB is the normalized histogram count of an image.

Furthermore, LH and HL sub-bands are decomposed by applying SVD to $U_{LH},\;S_{LH},\;V_{LH}$, and $U_{HL},\;S_{HL},\;V_{HL}$ sub-matrices, respectively [35]. SVD is applied to achieve robustness against filtering attack, addition of noise, histogram equalization, and geometric attacks. $S_{LH}$ and $S_{HL}$ are divided into 4×4 blocks. Watermark bits are embedded in selected blocks of $S_{LH}$ and $S_{HL}$. Block selection is done according to vector V i.e., the 4×4 blocks in $S_{LH}$ and $S_{HL}$ having corresponding lower entropy in 4×4 blocks of LH and HL, respectively, are selected for embedding and called $B_{LH}$ and $B_{HL}$. A low entropy block is selected for embedding to ensure higher imperceptibility. Watermark is partitioned into even and odd pixel vectors followed by encryption to obtain encrypted watermark vectors as explained in Section 3.2. Encrypted odd and even watermark pixels are embedded in $B_{LH}$ and $B_{HL}$, respectively. Thus, the number of $B_{LH}$ and $B_{HL}$ blocks is equal to P×Q/2. Embedding positions ($P_{1},P_{2}$) are determined randomly in the selected low entropy block based on a random sequence generated by a Blum–Blum–Shub (BBS) Pseudo random generator. BBS is deterministic in nature and has a one-way function; hence, it is difficult to break. BBS generates a Pseudo random number series by using initial seed value. For selection of seed value, the following conditions must be satisfied:

1. Select two prime numbers ‘a’ and ‘b’ and both are congruent to a(mod b).
2. Calculate the product of ‘a’ and ‘b’, say m. i.e., m=a×b.
3. Find integer as a co-prime for m, which is taken as the seed value ($Z_{n}$).

The formula for generating BBS Pseudo random series as shown in Equation (2):

(2) $$Z_{n}=Z_{n-1}^{2}\;mod\;m$$

where $Z_{n}$ is the *n*th term of BBS series and n is any positive integer. $Z_{1}$ is seed value and m=a×b.

The initial values required for BBS series generation are seed value (s) and m. The generation of BBS Pseudo random series is demonstrated in the following example:

Example of BBS approach:

  Let us consider that a = 11 and b = 19 are two large prime numbers.

m=11×19=209.

Selecting seed(s) as satisfying the condition as GCD of the s with the m is 1, i.e., GCD (3, 209) = 1

BBS series is 9, 81, 82, 36, 42, 92, 104, 157, 196, 169, 137, 168, …, *n*th term  

**Figure 2:**
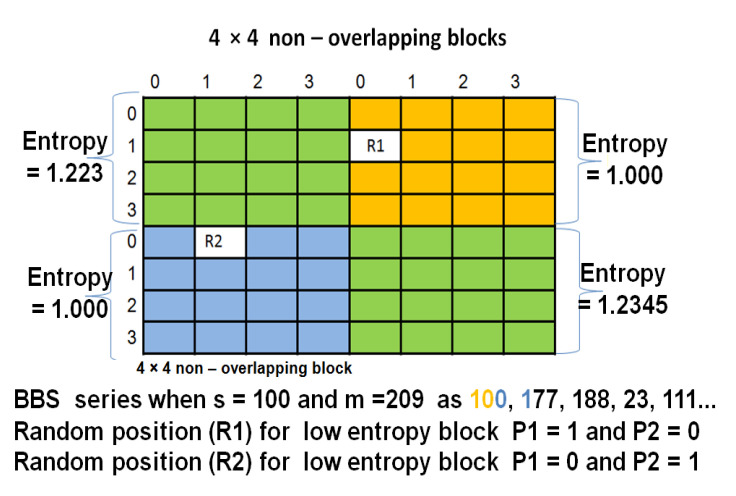
Determining the embedding position randomly in low entropy blocks.

Determining the embedding position in selected blocks is shown in Figure 2. After determining the embedding position, the encrypted watermark vectors ($V_{odd}^{1}$ and $V_{even}^{1}$) are embedded using adaptively generated ISF (α). Encrypted watermark vectors are generated by using the proposed encryption scheme, as explained in Section 3.2. α is generated by using the texture range filter elaborated in Section 3.3. Finally, SVD and IWT inverse transforms are applied to get a watermarked image.
**Algorithm 1** Watermark embedding.**Require:** 
Cover image (C), Watermark (W), m, s**Ensure:** 
Watermarked image ($C_{1}$)1:Apply 1-IWT to C, to obtain LL, LH, HL and HH. Select LH and HL for embedding.2:Divide LH and HL into 4×4 non-overlapping blocks.3:Find entropy for 4×4 non-overlapping blocks and store it in vector V.4:Apply SVD transform on LH and HL, to obtain $U_{LH},S_{LH},V_{LH},U_{HL},S_{HL},V_{HL}$. Select $S_{LH}$ and $S_{HL}$ for embedding.5:Select a block having a low entropy value $\left(B_{LH}\;and\;B_{HL}\right)$ for embedding.6:Encrypt the watermark image using the proposed encryption scheme7:Determine random embedding position $P_{1}\;\left(row\right)$ and $P_{2}\;\left(col\right)$, using a BBS generator in selected block for embedding.8:Generate α using the proposed intial scaling factor generation scheme9:For real time applications, skip Step 10 and move on to Step 11.10:Optimize α using GA or ABC or FO algorithm using a following fitness function:$Fitnessfunction=\frac{\left(PSNR\times SSIM\right)}{\alpha }+\frac{\left(NC\times BER\right)}{\alpha }$11:Embed $V_{odd}^{1}$ in $B_{LH}$ and $V_{even}^{1}$ in $B_{HL}$ using the following relation:$B_{LH}^{1}\left(P_{1},P_{2}\right)=B_{LH}\left(P_{1},P_{2}\right)+\alpha *V_{odd}^{1}$$B_{HL}^{1}\left(P_{1},P_{2}\right)=B_{HL}\left(P_{1},P_{2}\right)+\alpha *V_{even}^{1}$12:Apply inverse SVD and IWT to get the watermarked image ($C_{1}$).

**Figure 3:**
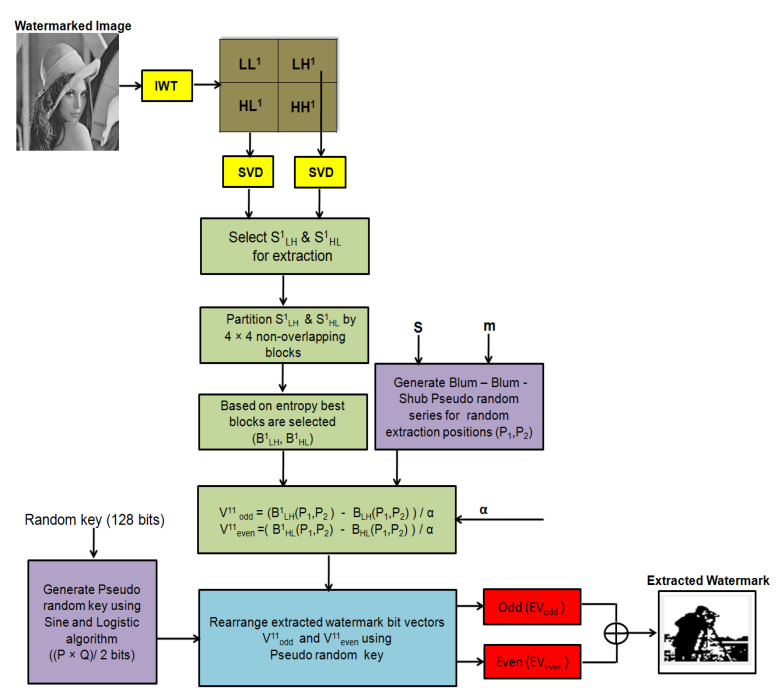
Block diagram for the watermark extraction process.

Watermark Extraction: Watermark extraction is the reverse process of watermark embedding. In the proposed scheme, the watermark is extracted from the watermarked image using secret keys (α, Random key, s and m) generated during the embedding process. The original cover image is not required for watermark extraction—therefore, the proposed scheme blind. The block diagram for the proposed watermark extraction is shown in Figure 3, and the algorithmic steps are provided in Algorithm 2. To extract the watermark, firstly, 1-IWT is applied on the received watermarked image to obtain $LL^{1}$, $LH^{1}$, $HL^{1}$ and $HH^{1}$ sub-bands. SVD transform is applied to $LH^{1}$, $HL^{1}$ sub bands to obtain three matrices each: $U_{LH}^{1},S_{LH}^{1},V_{LH}^{1}$, and $U_{HL}^{1},S_{HL}^{1},V_{HL}^{1}$. Furthermore, $S_{LH}^{1}$, $S_{HL}^{1}$ is divided into 4×4 non-overlapping blocks and low entropy blocks $B_{LH}^{1}$, $B_{HL}^{1}$ are selected for watermark extraction. The BBS Pseudo random series is generated, using the side information s, m (secret keys). Using BBS series, random positions ($P_{1}$, $P_{2}$) within $B_{LH}^{1}$, $B_{HL}^{1}$ blocks are determined for watermark extraction. Encrypted watermark vectors, $V_{odd}^{11}$ and $V_{even}^{11}$, are extracted from $B_{LH}^{1}$ and $B_{HL}^{1}$, respectively, as shown in step 6 of Algorithm 2. The pseudo random key ($PR_{key}$) is generated using random key ($R_{key}$ ) received as side information from the trusted third party. $PR_{key}$ is used to re-shuffle $V_{odd}^{11}$ and $V_{even}^{11}$ watermark vectors to obtain the decrypted odd and even watermark vectors ($EV_{even}\;and\;EV_{odd}$). Both odd and even watermark vectors are merged to get the extracted watermark (EW).
**Algorithm 2** Watermark extraction.**Require:** 
Watermarked image ($C_{1}$), m, s, Random key ($R_{key}$), α**Ensure:** 
Extracted Watermark (EW)1:Apply 1-IWT to $C_{1}$, to obtain $LL^{1}$, $LH^{1}$, $HL^{1}$ and $HH^{1}$ sub-bands2:Apply SVD to $LH^{1}$ and $HL^{1}$ sub-bands to obtain $U_{LH}^{1},S_{LH}^{1},V_{LH}^{1},U_{HL}^{1},S_{HL}^{1},V_{HL}^{1}$3:Divide $S_{LH}^{1}$ and $S_{HL}^{1}$ into 4×4 non-overlapping blocks.4:Select low entropy blocks $\left(B_{LH}^{1},B_{HL}^{1}\right)$.5:Determine watermark extraction positions $\left(P_{1}\;and\;P_{2}\right)$ using BBS Pseudo random series generated by using mands.6:Extract watermark vector from $S_{LH}^{1}$ and $S_{HL}^{1}$ using the steps below:$V_{odd}^{11}$←$(B_{LH}^{1}\left(P_{1},P_{2}\right)-B_{LH}\left(P_{1},P_{2}\right))/\alpha$$V_{even}^{11}$←$(B_{HL}^{1}\left(P_{1},P_{2}\right)-B_{HL}\left(P_{1},P_{2}\right))/\alpha$7:Generate a pseudo random key ($PR_{key}$) using $R_{key}$.8:Re-shuffle the extracted watermark vectors i.e., odd $\left(V_{odd}^{11}\right)$ and even $\left(V_{even}^{11}\right)$ vectors using $PR_{key}$.$EV_{odd}$←$PR_{key}$ · $V_{odd}^{11}$$EV_{even}$←$PR_{key}$ · $V_{even}^{11}$9:Combine $EV_{odd}$ and $EV_{even}$ to get the extracted watermark.EW←$EV_{odd}+EV_{even}$

### 3.2. Watermark Encryption and Decryption

In the proposed scheme, a symmetric cryptographic approach is used for watermark encryption. To encrypt the binary watermark, its pixels are partitioned into even $\left(V_{even}\right)$ and odd $\left(V_{odd}\right)$ position pixel vectors which is further shuffled according to a pseudo random key ($PR_{Key}$). Steps for watermark encryption are provided in Algorithm 3. Watermark partitioning and shuffling are explained below in detail.
**Algorithm 3** Watermark encryption.**Require:** 
Watermark (W) of size P×Q**Ensure:** 
Encrypted watermark vectors $V_{odd}^{1}\;and\;V_{even}^{1}$ of size P×Q/2, $R_{key}$, $PR_{Key}$ (P×Q /2)1:Partition W into even $\left(V_{even}\right)$ and odd $\left(V_{odd}\right)$ position pixel vectors.2:Generate 8-bit binary intermediate key $\left(IK_{B}\right)$.3:Generate 128 bit $R_{key}$ by hashing $\left(IK_{B}\right)$ using MD-5.4:Generate $PR_{Key}$ (( P×Q)/2) from $R_{key}$ using Algorithm 4.5:Shuffle $V_{odd}\;and\;V_{even}$ using $PR_{Key}$ to get the encrypted vectors $V_{odd}^{1}\;and\;V_{even}^{1}$.

**Figure 4:**
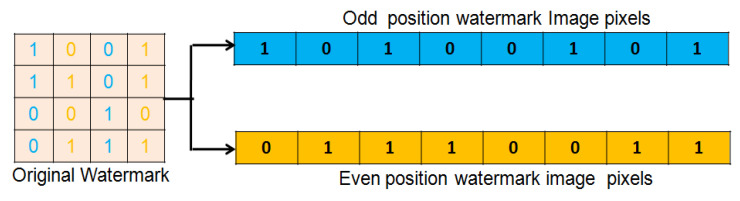
Partitioning even and odd position pixels of watermark image into $V_{even}$ and $V_{odd}$.

Watermark Partition: A watermark image is partitioned into even and odd position pixel vectors ( $V_{even}$ and $V_{odd}$) by scanning from top to bottom and left to right order in a raster scan line fashion. All even and odd position pixel values are appended to $V_{even}$ and $V_{odd}$, respectively, using Equation (3) and (4):

(3) $$V_{odd}\;\left(x\right)=\left\{\begin{array}{c} W(row,col),\;if\;mod(col,2)\neq 0\\ \;\;Otherwise,\;ignored, \end{array}\right.$$

(4) $$V_{even}\;\left(y\right)=\left\{\begin{array}{c} W(row,col),\;if\;mod(col,2)=0\\ Otherwise,\;ignored, \end{array}\right.$$

where W (row, col) is the original watermark image. $V_{odd}\left(x\right)$ and $V_{even}\left(y\right)$ are odd and even position pixels of the watermark image, respectively. Further illustrations of watermark partitioning are provided in Figure 4.

Watermark Shuffling: Vectors $V_{even}$ and $V_{odd}$ are shuffled by using a pseudo random key ($PR_{key}$) which is generated from a 128 bit $R_{key}$. To ensure that $R_{Key}$ cannot be cracked, a unique binary intermediate key ($IK_{B}$) is used as an initial parameter. $IK_{B}$ is generated by using the following relations:

(5) S=ceil(sum(μLL,μLH,μHL,μHH,∑W))

$$IK=\left\{\begin{array}{c} S,\;\;\;\;\;\;\;\;\;\;\;\;\;if\;S\leq 255\\ S\;\%255,\;if\;S>255 \end{array}\right.$$

$$IK_{B}=Binary\left(IK\right)$$

where µLL, µLH, µHL and µHH are mean of the IWT sub-bands LL, LH, HL and HH, respectively. ∑W is sum of binary watermark image bits.

Numerical explanation for $IK_{B}$ generation:  

Let us consider, µLL = 124.0369, µLH = −0.0337, µHL = 0.11666 and µHH = 0.0144 and $\sum_{W}$ = 2312 S = ceil(124.0369 + −0.0337 + 0.1166 + 0.0144 + 2312 ) = 2437

IK = 2437 % 255 = 142

$IK_{B}$ = Binary equivalent of IK = Binary(142) = 10,001,110 (8bits)  

$R_{key}$ acts as the secret key for the watermark encryption and decryption process. It can be shared between the sender and receiver as a secret key. The $R_{key}$ is generated by hashing the unique intermediate key $\left(IK_{B}\right)$. Hash functions are highly secured approaches for the generation of authentication codes for images. Researchers have used different hash functions like MD-5, SHA-1, SHA-256, and SHA-512 for generating secured authentication code. Owing to its low computational cost and high security, MD-5 is used in the proposed scheme. $IK_{B}$ is input to MD-5 for generating 128 bits $R_{key}$ as shown in Equation (6):

(6) $$RandomKey\left(R_{key}\right)=MD5\left(IK_{B}\right).$$

**Figure 5:**
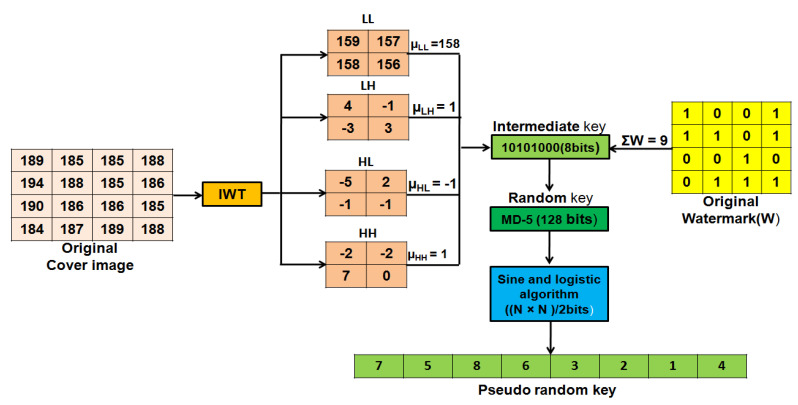
Pseudo random key generation.

**Figure 6:**
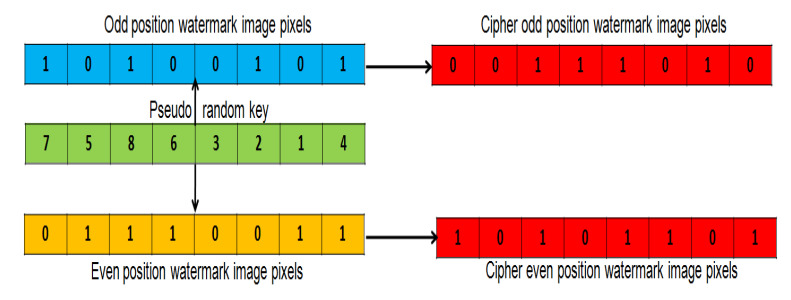
Watermark shuffling at the sender’s end.

$PR_{key}$ of size ((P×Q)/2) is generated from $R_{key}$ using Sine and Logistic algorithm as explained in Algorithm 4. The process of Pseudo random key generation is illustrated with an example in Figure 5. For the generation of a cipher watermark, the vectors of odd $\left(V_{odd}\right)$ and even $\left(V_{even}\right)$ watermark pixels position are shuffled with respect to $PR_{key}$. Watermark shuffling at the sender end is illustrated in Figure 6.
**Algorithm 4** Pseudo random key generation from Random key.**Require:** 
Random key (128 bits)**Ensure:** 
Pseudo Random key ((P×Q)/2 )1:Initial conditions $a_{0}$, $b_{0}$, $S_{1}$ and $S_{2}$ are generated as follows:
$$a_{0}=\sum_{K=1}^{20}\frac{Randomkey(K)}{2^{K}}$$
$$b_{0}=\left(\;\sum_{K=21}^{50}\frac{Randomkey(K)}{2^{K-52}}\right)\;mod1$$
$$\begin{array}{cc} S_{1}=\; & (\;\sum_{K=1}^{8}Randomkey\left(K\right)\times 2^{K}\;\\ & +\sum_{K=25}^{76}\frac{Randomkey(K)}{2^{K-24}}+b_{0})\;mod10+10 \end{array}$$
$$\begin{array}{cc} S_{2}=\; & (\;\sum_{K=121}^{128}Randomkey\left(129-K\right)\times 2^{K}\;\\ & +\sum_{K=77}^{128}\frac{Randomkey(K)}{2^{K-76}}\times S_{1})\;mod10+10 \end{array}$$
$$a_{0}=\left(\;a_{0}+S_{2}\right)\;mod1$$2:The initial values $a_{0},S_{1}\;and\;b_{0},S_{2}$ are used for the logistic and sine map, respectively. The maps are iterated P×Q times, whereby the random sequences are stored as matrices LF and SF of size P×Q as
$$\begin{array}{cc} LF=\; & mod(\;(\;power\left(S_{1},2\right)\times a_{0}\;\\ & \times \left(\;1-S_{1}*a_{0}\right)\;+\frac{S_{1}}{a_{0}})\;,1)\; \end{array}$$
$$\begin{array}{cc} SF=\; & mod(\;(\;S_{2}*sin\left(\;180*S_{2}*b_{0}\right)\;\;\\ & +\frac{S_{2}}{b_{0}},1)\; \end{array}$$3:Based on these intermediary matrices, a final Pseudo random key $PR_{Key}$ is then calculated as
$$\begin{array}{c} PR_{key}=\sum_{k=1}^{P}\sum_{l=1}^{Q}\left(\;\left(\;\left(\;LF\left(k,l\right)+SF\left(k,l\right)\right)\;\right)\;mod\;10\right)\; \end{array}$$
where *LF(k, l)* and *SF(k, l)* are the elements of the *LF* and *SF* matrices, respectively, while *k* and *l* denote the row and column of *LF*. The resulting matrix $PR_{key}$ is Pseudo random key in the range of $\left[1,\frac{\left(P\times Q\right)}{2}\right]$, where P×Q is the size of the watermark image.

**Figure 7:**
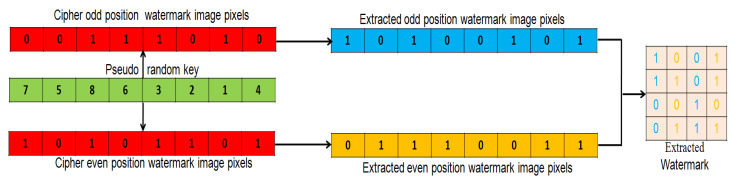
Watermark decryption at the receiver end.

Watermark Decryption: For watermark decryption, the Sine and Logistic algorithm is applied on the 128 bit $R_{Key}$ (received secretly from the sender) to generate $PR_{Key}$ at the receiver end. The extracted watermark vectors $V_{even}^{11}$ and $V_{odd}^{11}$ are re-shuffled according to $PR_{Key}$ to obtain $EV_{even}$ and $EV_{odd}$. The values of $EV_{even}$ and $EV_{odd}$ are populated to the corresponding even and odd pixel positions in raster fashion to obtain the extracted watermark (decrypted). Watermark decryption at the receiver end is shown in Figure 7.

Security analysis: The proposed scheme ensures an efficient watermark encryption in two ways. Firstly, the watermark is partitioned into two vectors followed by the shuffling of vectors corresponding to the $PR_{Key}$. $PR_{Key}$ is generated from the 128 bit $R_{Key}$, which is unique to each cover and watermark image. $R_{Key}$, Sine and Logistic algorithm together can only generate $PR_{Key}$ for watermark encryption and decryption. Even though the random key is small (128 bits), if the attacker owns the random key without knowing the algorithm, it is not possible to decrypt the watermark. Due to the initial condition sensitivity of $IK_{B}$, any changes to the intermediate key will lead to an entirely new $R_{Key}$. Hence, this provides two-fold security to the secret key.

### 3.3. Initial Scaling Factor Generation and Optimization

**Figure 8:**
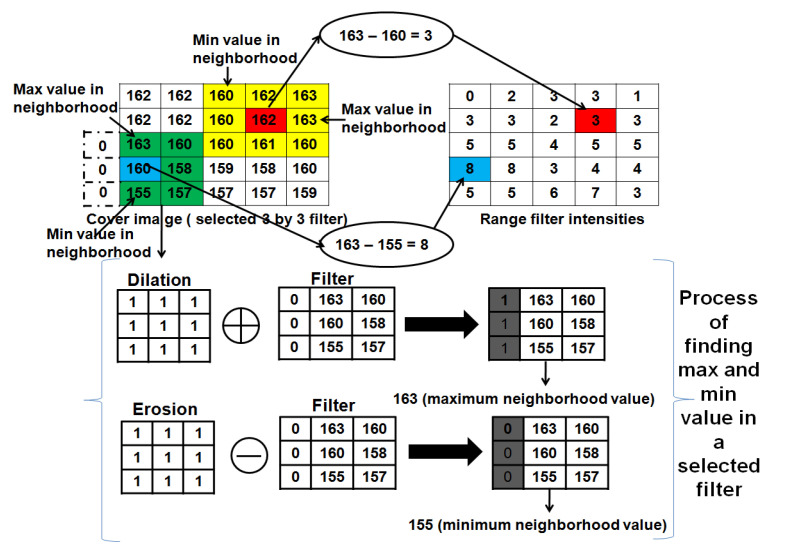
Range filter.

**Figure 9:**
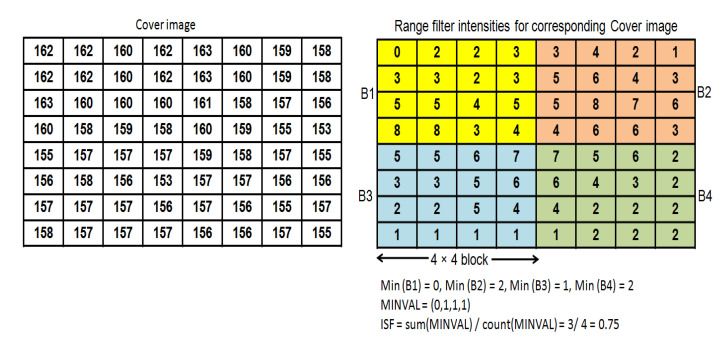
ISF generation from the cover image.

The visual quality and robustness of watermarking scheme largely depends on embedding strength parameter (α). Until now, the majority of DIW schemes choose random ISF (α) for watermark embedding and the extraction process. Choosing the same random ISF for all image modalities may degrade visual quality. To ensure higher visual quality, the proposed scheme generates ISF adaptively from the cover image using image range texture characteristics. The algorithmic steps for adaptive ISF generation is presented in Algorithm 5. The fuzzy based image texture range filter characteristic is used for generation of ISF adaptively from the cover image. The image range filter defines a neighborhood around the pixel of interest and calculates the statistics for that neighborhood. If the intensities in the image range has more variability, this indicates that there is a distinguished foreground and background in the image. Adaptive generation of ISF is computationally inexpensive. In the proposed scheme, less variability regions are selected for embedding to achieve higher visual quality. For ISF generation, a 3×3 neighborhood filter function is used. The relation used for generation of range values is in Equation (7):

(7) Range=Maxval−Minval

where Maxval is the maximum intensity and Minival is the minimum intensity values of the selected 3×3 filter. The proposed scheme generates Maxval and Minval from the selected 3×3 filter using morphological operations called dilation and erosion, respectively. The dilation operator results in a maximum value and the erosion operator results in a minimum value in a selected filter based on their mask filter. Generation of range filter intensities of the 3×3 filter is illustrated with the help of an example in Figure 8 using dilation and erosion operators. Algorithmic steps for adaptive generation of ISF from the cover image are shown in Algorithm 5. Furthermore, the process of ISF generation is exhibited with an example in Figure 9.
**Algorithm 5** Initial Scaling Factor (α) generation.**Require:** 
Cover image (C)**Ensure:** 
ISF (α)1:Find range filter (R) values for C using 3×3 filter2:Partition R into 4×4 non-overlpping blocks.3:Find minimum value in each block and save it in MINVAL.4:Average of MINVAL is (α) for C.

If computational cost is not the major concern, then ISF can be further optimized for achieving higher watermarking characteristics. Nature Inspired Optimization (NIO) algorithms such as Genetic algorithm (GA), Artificial Bee Colony (ABC), or Firefly optimization (FO) are proposed for optimizing ISF. Optimization algorithms are used to find solutions that maximize or minimize some study parameter. NIO algorithms are stochastic metaheuristic based evolutionary algorithms developed by the inspiration of nature suitable for larger search space. Researchers have proposed a number of NIO optimization algorithms based on swarm intelligence (ABC, PSO, ACO, Firefly, etc.) and based on genetic behavior (GA, etc.). Among all these, GA, ABC and FO have good exploration and exploitation capabilities to reach the global optimum at lower time intervals. In the proposed scheme, GA, ABC and FO metaheuristic based evolutionary algorithms are adapted for ISF optimization. The fitness function used for obtaining optimized ISF (α) is shown in Equation (8):

(8) $$Fitnessfunction=\frac{\left(PSNR\times SSIM\right)}{\alpha }+\frac{\left(NC\times BER\right)}{\alpha }$$

## 4. Experimental Results and Discussion

**Figure 10:**
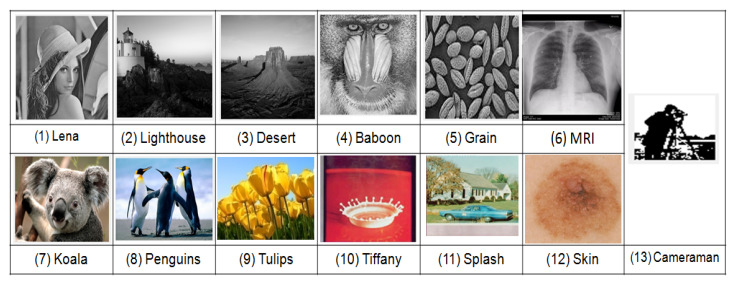
Grayscale cover images (**1**–**6**), color cover images (**7**–**12**) and binary watermark (**13**).

This section presents experimental results to demonstrate the performance of the proposed scheme in terms of various watermarking characteristics like imperceptibility, robustness, security, embedding rate and computational time using MATLAB 2014b with an Intel i5 processor, 2.00 GHz, 4 GB RAM. Test cover images have been taken from USC-SIPI [36] and the UCID [37] dataset as shown in Figure 10. For convenience of the representation, 12 images have been taken for visualization. The size of cover image (gray-scale and color) is 512×512 and watermark (binary) is 64×64.

### 4.1. Imperceptibility Test

**Figure 11:**
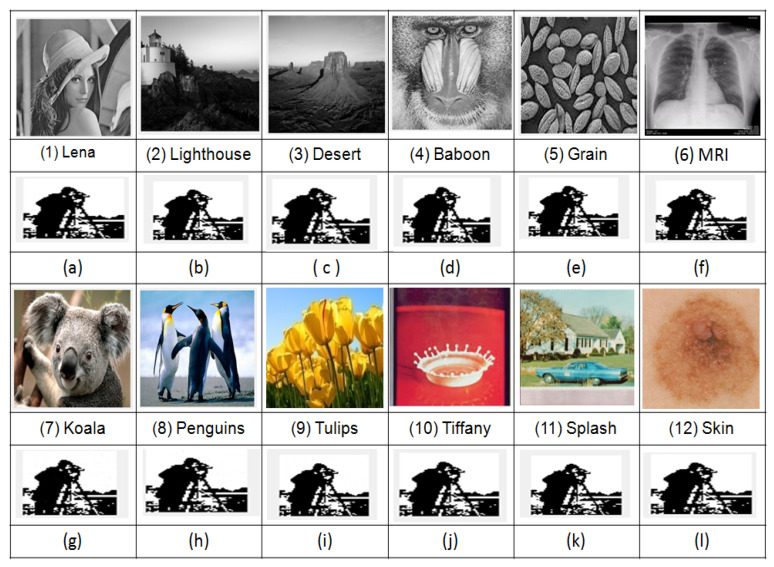
Watermarked images: Grayscale (**1**–**6**), Color (**7**–**12**) and extracted watermark images (**a**–**l**).

Imperceptibility is an important characteristic for all DIW applications. For a good watermarking scheme, the photographic quality of the cover and watermarked images should be almost the same. To evaluate the imperceptibility of the proposed scheme, subjective (qualitative) and objective (quantitative) analysis is carried out. Test cover images and corresponding watermarked images are shown in Figure 10 and Figure 11, respectively. Subjective analysis of the cover image and its corresponding watermarked images exhibit no significant change to HVS (Human Visual System). This observation can be affirmed from images in Figure 10 and Figure 11.

Furthermore, objective analysis for the imperceptibility performance is analyzed using Mean Square Error (MSE), Peak Signal-to-Noise Ratio (PSNR) and Structural Similarity Index Metric (SSIM). MSE is a statistic measure to estimate the imperceptibility of watermarking scheme from the square of Euclidian distance. It estimates the error between the original and watermarked image. The mathematical relation for MSE shown in Equation (9):

(9) $$MSE=\frac{1}{M\times N}\sum_{r=1}^{M}\sum_{c=1}^{N}{\left[C\left(r,c\right)-C_{1}\left(c,r\right)\right]}^{2}$$

where *C* is Cover image, and $C_{1}$ is a watermarked image. PSNR is a good measure of pixel difference between two images. It is calculated using relations in Equation (10):

(10) $$PSNR=10\;log\;10\left(\frac{255^{2}}{MSE}\right)$$

SSIM is a measure of three image features as luminance (ll), brightness (bb), structure (ss), and it is in accordance with the HVS. The mathematical relation for SSIM is shown in Equation (11):

(11) $$SSIM=\left[ll\left(C,C_{1}\right).\;bb\left(C,C_{1}\right).\;ss\left(C,C_{1}\right)\right]$$

$$ll\left(C,C_{1}\right)=\frac{2C_{mean}*{C_{1}}_{mean}}{{C^{2}}_{mean}+{{C_{1}}^{2}}_{mean}}$$

$$bb\left(C,C_{1}\right)=\frac{2C_{var}*{C_{1}}_{var}}{{C^{2}}_{var}+{{C_{1}}^{2}}_{var}}$$

$$ss\left(C,C_{1}\right)=\frac{{CC}_{1cvar}}{C_{var}+{C_{1}}_{var}}$$

where $C_{mean},\;{C_{1}}_{mean}$ as a mean of $C,C_{1},$ respectively; ${C^{2}}_{var}$, ${{C_{1}}^{2}}_{var}$ as variance of $C,C_{1}$ and ${CC}_{1cvar}$ as co-variance of $C\;and\;C_{1}$.

Experiments are performed on different cover images with adaptively generated ISF (α). PSNR, SSIM, MSE and ISF values for the images shown in Figure 10 are tabulated in Table 1. It can be observed from Table 1 that, for all grayscale images, PSNR is above 51 dB and the average is 52.16 dB. For color images, PSNR is above 56 dB and the average is 57.89 dB. For both grayscale and color images, PSNR is greater than the threshold value of 37 dB and the average error rate is minimal as the MSE value is low. SSIM for grayscale images are above 0.9600 and the average is 0.9754, whereas SSIM for color images is above 0.9991, and the average is 0.9988. For all test cover images, SSIM is approaching the ideal value of 1. The proposed scheme shows higher performance for “Tulips” image having PSNR = 60.85 dB with SSIM = 1. Further imperceptibility performance of the proposed scheme is evaluated for 50 images of different modalities taken from the USC- SIPI dataset [36], and the imperceptibility performance (PSNR, SSIM, MSE) is provided in Table 2. From the table, it can be observed that, for 50 images, PSNR varies between 51.42 dB (for the image Sail boat) to 58.47 dB (for the image Pixel ruler) and an average of 51.55 dB. MSE varies between 0.5907 (for the image Stream and Bridge) to 0.0899 (for the image Jelly bean) and an average of 0.3813. SSIM varies between 0.6337 (for the image Resolution chart) to 0.9999 (for the image Grass) and an average of 0.9767. It is observed that, for the image “Pixel Ruler”, the proposed scheme shows higher PSNR (58.47 dB) and low MSE (0.0924). Subjective and objective analysis of imperceptibility shows that the proposed scheme has high imperceptibility for various image modalities. It improves further by using optimized ISF.

The imperceptibility performance of the proposed scheme using optimized ISF is also analyzed using GA, ABC and FO algorithms and presented in Table 3. Comparing the result in Table 1 and Table 3, it can be observed that PSNR and SSIM for grayscale and color images increased after optimization. Using GA, the average value of PSNR for grayscale images increased from 52.16 dB to 52.95 dB and, for color, it is improved from 57.89 dB to 58.42 dB. In addition, SSIM increased from 0.9654 to 0.9989 and 0.9988 to 0.9989, for grayscale and color images, respectively. Improved performance is also observed by using ABC and FO, but GA exceeds in improving the imperceptibility performance. From the above discussion, it can be inferred that the proposed scheme has high imperceptibility for grayscale as well as color images of different modalities. Further imperceptibility improves by using optimized scaling factor.

### 4.2. Robustness

Robustness performance for the proposed watermarking scheme is evaluated under zero and various common attacks by using Normalized Correlation (NC) and Bit Error Rate (BER) as performance metrics. NC is a good measure for robustness that gives normalized correlation between original and extracted images in terms of direction and strength relationship. BER measures pixel level difference between original and extracted images. The relation for NC and BER is provided in Equations (12) and (13), respectively:

(12) $$NC=\frac{\sum_{r=1}^{P}\sum_{c=1}^{Q}{\left[W\left(r,c\right)-W^{1}\left(r,c\right)\right]}^{2}}{\sqrt{\left[\sum_{r=1}^{P}\sum_{c=1}^{Q}W{\left(r,c\right)}^{2}\right]}\times \sqrt{\left[\sum_{r=1}^{P}\sum_{c=1}^{Q}W^{1}{\left(r,c\right)}^{2}\right]}}$$

where W(r,c) t and $W^{1}\left(r,c\right)$ are original and extracted watermarks:

(13) $$BER=\frac{EB}{TB}$$

$$EB\;=\left\{\begin{array}{cc} counter+1 & if\sum_{r=1}^{P}\sum_{c=1}^{Q}W\left(r,c\right)\neq W^{1}\left(r,c\right)\\ 0 & otherwise \end{array}\right.$$

TB=P×Q

where EB represents the number of incorrectly decoded bits in extracted watermark, and TB represents total number of bits and initial value of counter = 0.

#### 4.2.1. Adaptive ISF

**Figure 12:**
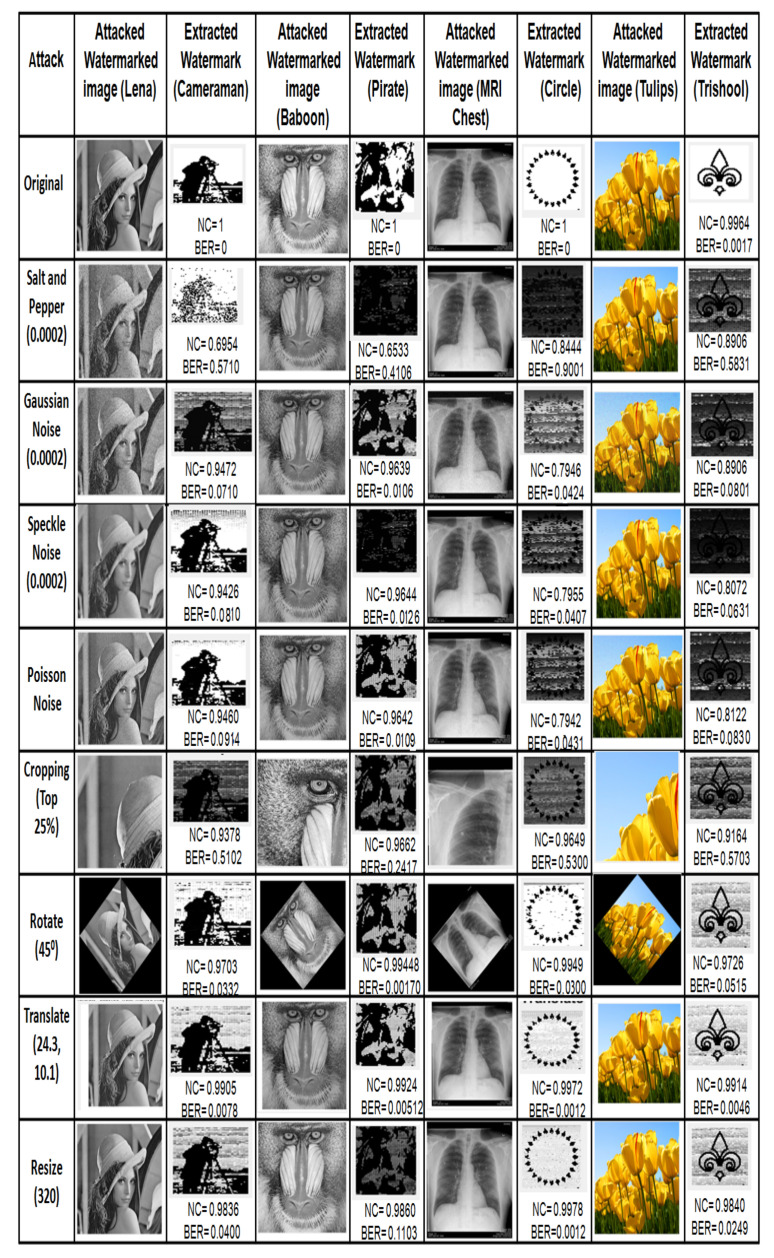
Attacked watermarked images and corresponding extracted watermark images with NC and BER values under different attacks.

**Figure 13:**
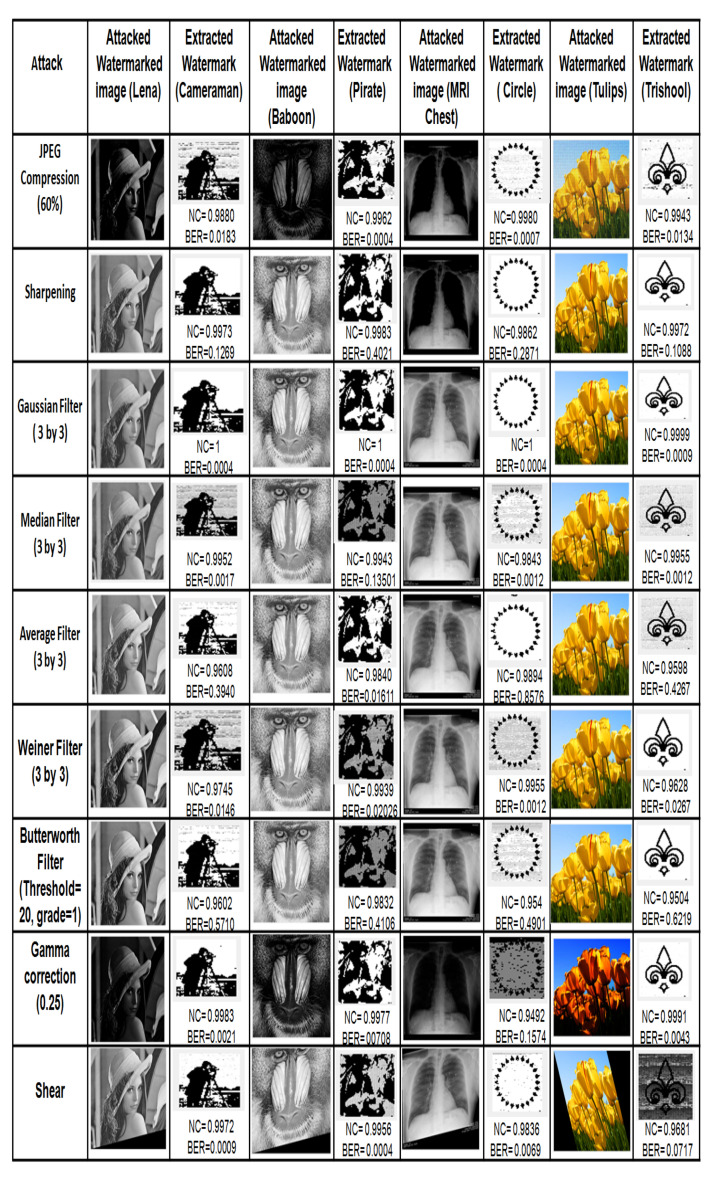
Attacked watermarked images and corresponding extracted watermark images with NC and BER values under different attacks.

Robustness of the proposed scheme using adaptive ISF for the test cover images Figure 10 under zero attacks is presented in Table 1, and the corresponding extracted watermark is shown in Figure 11. For all grayscale images, NC and BER are equal to ideal values 1 and 0, respectively, as observed from Table 1. Whereas, for all color images, average NC is 0.9940, and the average BER is 0.0115. Furthermore, it is observed from Table 2 that NC and BER, for 50 images of different modalities, under zero attacks are also equal to an ideal value. These observations implicate that the watermark is successfully extracted under zero attack. Further robustness performance of the proposed scheme is examined for different cover images (Lena, Baboon, MRI Chest, Tulips) and watermark (Cameraman, Pirate, Circle, Trishool) images under common image processing attacks. Attacked watermarked image and corresponding extracted watermark with NC and BER are presented in Figure 12 and Figure 13, where it can be observed that the proposed scheme successfully extracts the watermark under filtering, geometrical and compression attacks from all cover images. For noise attacks, the proposed scheme extracts watermarks with little distortion. The above sampled consequences indicate that the proposed scheme is robust against the majority of attacks.

#### 4.2.2. Optimized ISF

ISF optimization using GA, ABC and FO is suggested for improved performance of the proposed scheme. The robustness performance of the proposed scheme using optimized ISF is also evaluated. NC and BER under zero attacks for different test cover images are shown in Table 4. NC and BER values are equal to ideal values for all grayscale images under zero attacks as can be studied from Table 4. For color images, from Table 4, it can be observed that the average NC value has increased from 0.9940 (with adaptive ISF) to 0.9998 (with optimized ISF) and BER decreased from 0.0115 to 0 using GA. By using ABC, NC increased from 0.9940 to 0.9995 and BER reduced from 0.0115 to 0. With FO, NC improved from 0.9940 to 0.9991 and BER reduced from 0.0115 to ideal value 0. After optimization of ISF, for the images “Penguins” and “Tulips”, NC increased from 0.9992, 0.0919 to 1. For the images “Koala”, “Flash” and “Skin”, BER reduced from 0.0022, 0.229, 0.0014 to ideal value 0. From this discussion, it can be observed that, for color images, robustness increased significantly after optimization. Further robustness of the proposed scheme is evaluated for “Lena” image under different attacks using adaptive and optimized ISF and compared in Table 5. From Table 5, it can be seen that NC values for the majority of attacks are higher for ISF optimized by using GA, hence making the watermark more robust against common attacks. Thus, optimization can be used to improve the robustness of the proposed scheme. In addition, ISF optimization using GA is more pertinent for the proposed scheme.

### 4.3. Security Test

**Figure 14:**
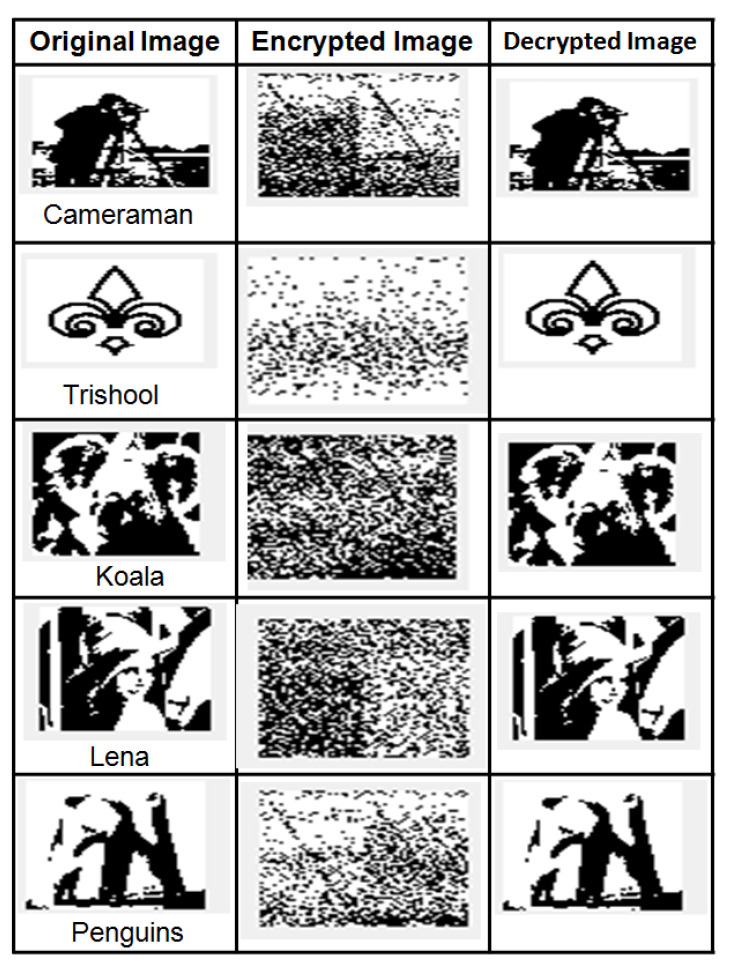
Original, Encrypted and Decrypted watermark images.

Watermark security is one of the important requirements of DIW schemes. Encrypted and decrypted watermark images obtained by using the proposed scheme are shown in Figure 14. Subjective analysis from Figure 14 indicates that encrypted images are very different from the original image, whereas the decrypted images are similar to the original image.

To study the effectiveness of the proposed encryption and decryption scheme, Correlation Coefficient (CC) is used. CC is a commonly used statistical measure for assessing the degree of linear relation between two images. The mathematical relation for CC is shown in Equation (14):

(14) $$CC=\frac{\sum_{r=1}^{P}\sum_{c=1}^{Q}\left(W_{r,c}-\mu \left(W\right)\right)\left(W_{r,c}^{1}-\mu \left(W^{1}\right)\right)}{\sqrt{\sum_{r=1}^{P}\sum_{c=1}^{Q}\left(W_{r,c}-\mu \left(W\right)\right)}\times \sqrt{\sum_{r=1}^{P}\sum_{c=1}^{Q}\left(W_{r,c}^{1}-\mu \left(W^{1}\right)\right)}}$$

where W, $W^{1}$ are the two images. μ(W) and $\mu (W^{1})$ are the mean values of W, $W^{1}$ images. Two identical images have CC = 1, whereas two completely uncorrelated images have CC = 0. If the two images are completely anti-correlated, then CC = −1. The security of the proposed scheme is studied using CC in terms of Horizontal (row), Vertical (column), and Diagonal (Cross) directions. CC is examined for binary watermark images shown in Figure 14, and performance is tabulated in Table 6. CC between original-encrypted images and CC between original-decrypted images can be studied in Table 6. CC between original-encrypted images in horizontal, vertical and diagonal directions for all test images are close to zero, indicating that encrypted images are uncorrelated to the original image. From Table 6, it can be observed that CC of original-decrypted images in horizontal, vertical and diagonal are equal to 1, indicating that original and decrypted images are highly correlated and are completely the same. From this, it can be claimed that the proposed scheme generates a strong cipher image and successfully decrypts the original image.

Furthermore, sensitivity of the random key has been evaluated by changing its bits. Even one bit of difference in the pseudo random key leads to unsuccessful decryption of the extracted watermark. To evaluate the random key sensitivity, CC is calculated between two encrypted images by changing one bit in random key and tabulated in Table 7. For all cases, CC in horizontal, vertical and diagonal directions is negative as in Table 7. It shows that, with one bit of change, random key forms are completely different than cipher images. From this discussion, it is evident that the random key is highly secured.

### 4.4. Computational Time

The time required for watermark embedding and extractions process in the DIW scheme is termed as computational time. The computational time of the major algorithmic steps of the proposed scheme is provided in Table 8. Considering only major algorithmic steps and the most expensive operations, the proposed adaptive watermarking scheme has cubic time complexity as shown below:

(15) $$\begin{array}{c} Computational\;time=O\left(2MN\right)+O\left(2MN\right)+O\left(2MN^{2}+2N^{3}\right)\\ +O\left(2M2N\right)+O\left(MN\right)+O\left(MN\right)+O\left(log\left(logm\right)\right)=O\left(N^{3}\right) \end{array}$$

Table 9 shows the computational time (in seconds) for the proposed scheme. From Table 9, it can be studied that the average embedding time for grayscale and color images is 1.527 s and 1.382 s, respectively, whereas the average extraction times for grayscale and color images are 0.995 s and 0.870 s, respectively. For all images, extraction time is less than the embedding time since pseudo random key generation is not done during extraction. There is no significant difference between embedding and extraction time for grayscale and color images. Computational time of the proposed scheme is optimum as the watermark is embedded and extracted in less than 2 s and 1 s, respectively.

### 4.5. Comparative Study

The performance of the proposed scheme is further validated by comparing it with recent state-of- the-art DIW schemes [18,23,27,28,31,32] in terms of imperceptibility, robustness, embedding rate, security, and fitness function. Performance comparison has been done with a non-blind scheme proposed by Ansari and Pant [18] and blind schemes proposed by Moeinaddini [31], Singh and Bhatnagar [32], Sharma and Mir [27], and Zainol et al. [23]. The scheme proposed in [18] uses PC for embedding watermark bits in the DWT-SVD domain. Arnold map is used for security, and scaling factor is optimized with ABC. This scheme suffers from low imperceptibility, robustness, and security. The Hadmaard transform based scheme is proposed in [31]. This scheme uses DDFA for scaling factor optimization. Watermark is embedded by adjusting Hadmaard coefficients, and it overlooked watermark security. The scheme proposed in [32] uses an LWT based adaptive embedding approach using a d-sequence. Arnold map is used for watermark security. This scheme has a lower embedding rate than other schemes [18,23,27]. A DCT based adaptive embedding scheme is proposed in [27], and it generates embedding blocks using an LGBA machine learning approach. This scheme also ignored watermark security. The scheme proposed in [23] uses IWT-SVD transform, and it shows lower NC values for rotation, JPEG compression, Gaussian filter, and Median filtering attacks. This scheme has high imperceptibility, security, and embedding rate at the cost of robustness. Table 10 shows a comparative study of watermarking parameters for the proposed scheme and schemes in comparison.

**Figure 15:**
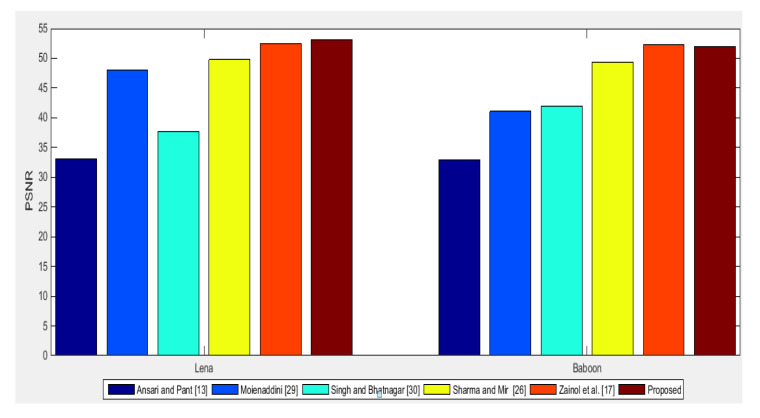
PSNR of the proposed scheme and the schemes in comparison [18,23,27,31,32] for Lena and Baboon test cover images.

**Imperceptibility Comparison:** Imperceptibility performance of the proposed scheme is compared with schemes proposed in [18,23,27,31,32] using PSNR. For comparative study, two popular test images “Lena” and “Baboon” are considered. Figure 15 shows PSNR values of the proposed scheme and schemes in [18,23,27,31,32] for “Lena” and “Baboon”. The PSNR values in Figure 15 indicate that the proposed scheme has higher imperceptibility than schemes in [18,23,27,31,32] with a higher/equal embedding rate. From this, it can be claimed that the proposed scheme has higher imperceptibility than schemes in comparison.

**Figure 16:**
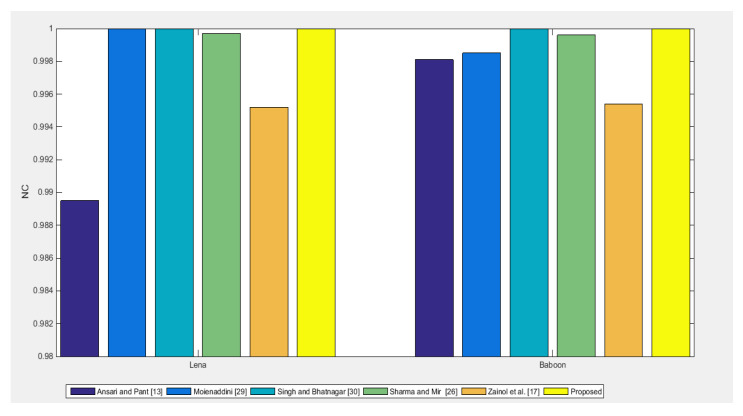
NC for the proposed scheme and watermarking schemes proposed in [18,23,27,31,32] (under zero attacks) for Lena and Baboon cover images.

**Figure 17:**
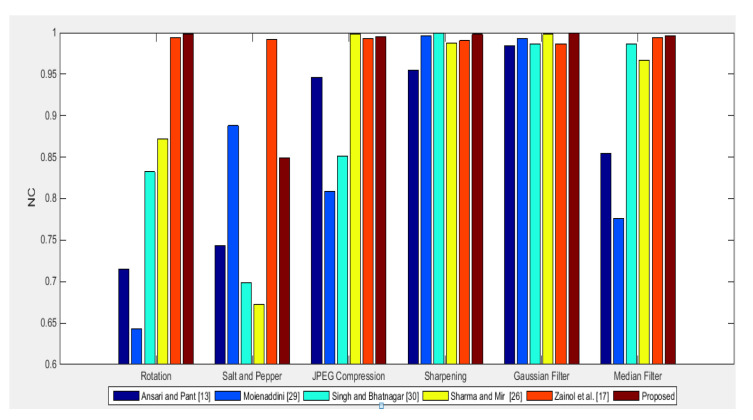
lNC for the proposed scheme and state-of-the-art schemes [18,23,27,31,32] under attacks for Lena cover Image.

Robustness Comparison: Robustness performance of the proposed scheme is compared with schemes in [18,23,27,31,32] using NC, under zero attacks as shown in Figure 16. The NC values of the proposed scheme are ideal/higher than other schemes [18,23,27,31,32] in comparison for “Lena” and “Baboon” images as can be seen in Figure 16. Further robustness performance is compared under image processing attacks and shown in Figure 17. The proposed scheme is more robust for rotation, sharpening, Gaussian filter, and median filter attacks against other schemes [18,23,27,31,32] in comparison. [18,23,27,31,32]. For JPEG compression, the proposed scheme is more robust than schemes in [18,23,31,32] and shows almost similar performance to the scheme in [27]. For a salt and pepper attack, the proposed scheme performs better than the schemes in [18,27,32] but lags behind in comparison to the scheme in [31]. The majority of the attacks in the proposed scheme are more robust and show almost equal performance to schemes in [23,27]. From this, it can be claimed that the proposed scheme is more robust than the state-of-the-art [18,23,27,31,32] schemes.

Embedding rate: The embedding rate of the proposed adaptive embedding scheme is analyzed in this section. The number of watermark bits embedded in the cover image is termed as the embedding rate of the watermarking scheme. The embedding rate of the proposed scheme is calculated using Equation (16) as shown below:

(16) $$Embedding\;\;rate=\frac{Total\;number\;of\;watermark\;bits}{Total\;number\;of\;cover\;image\;pixels}bpp$$

While calculating the embedding rate, the size of cover and the watermark are considered. The proposed scheme embedding rate is calculated using Equation (16) shown below:

Totalnumberofcoverimagepixels=512×512=262144pixels

Totalnumberofwatermarkbits=64×64=4096bits

$$Embedding\;rate\;=\frac{4096}{262144}=0.015625\;bpp$$

The proposed scheme has an embedding rate of 0.015625 bpp. The embedding rate of the proposed scheme is higher than the scheme proposed in [32] and equal to schemes proposed in [18,27,31]. The proposed scheme providing high imperceptibility, robustness and security than the scheme proposed in [23] but under-performance in terms of embedding rate. The scheme proposed in [23] has a higher embedding rate at the cost of lower imperceptibility, robustness and security. Even though the embedding rate of the proposed scheme is lower than [23], still, in comparison to the state-of-the-art schemes, the proposed scheme shows higher imperceptibility, robustness and security for different image modalities.

Security comparison: Schemes proposed in [27,31] have paid less attention to watermark security, whereas schemes in [18,32] use Arnold maps for watermark security, which can be easily cracked and thus provide low security. Generating a chaotic map scheme [23] ensures security but suffers from hyper tuning issues. Using two-level adaptive embedding and symmetric cryptographic approaches security is achieved in the proposed scheme. First, the watermark is partitioned into two parts and then encrypted using a pseudo random key. By the use of mathematical theory and the algorithm, the proposed scheme generates a Pseudo random key. It is difficult to predict the Pseudo random key generated by the proposed scheme by an attacker. Secondly, encrypted watermark is embedded at a random position selected by using BBS in low entropy blocks. This further makes the fort strong and non-invadable. When compared to other schemes in [18,23,32], the proposed scheme has a highly secured encryption approach. From the above discussion, it can be claimed that the proposed scheme provides higher watermark security than all other comparative schemes [18,23,27,31,32].

Fitness function: Designing a proper fitness function for NIO algorithms is very important. To demonstrate the effectiveness of the proposed fitness function, imperceptibility and robustness performance comparison are done with the scheme proposed in [28] where scaling factor (α) is optimized by using ABC [18]. Minimized fitness function derived from watermarking characteristics (PSNR, SSIM, NC, and BER) is used in [28] as shown in Equation (17):

(17) $$f=\frac{\left(PSNR\times SSIM\right)}{\alpha }+\frac{\left(NC\times BER\right)}{\alpha }-1$$

where scaling factor (α) is an initial random value, whereas, in the proposed scheme, maximized fitness function is used as shown in the following equation:

(18) $$f=\frac{\left(PSNR\times SSIM\right)}{\alpha }+\frac{\left(NC\times BER\right)}{\alpha }$$

where scaling factor (α) is the initial seed value generated adaptively from the image. Since (α) is image dependent, the maximized fitness function is used. The maximum fitness value is used to achieve high watermarking characteristics.

Imperceptibility performance of the proposed scheme is compared with the scheme in [28] by optimizing scaling factor (α) using ABC and GA in Table 11. Here, it can be observed that, for all images, the proposed scheme has higher PSNR and SSIM than the scheme in [28] for ABC and GA. For all color images, remarkable improvement can be observed by the proposed scheme over [28] with ABC as well as GA from Table 11. Hence, it can be claimed that the proposed scheme has higher imperceptibility as compared to [28]. Robustness of the proposed scheme and the scheme in [28] with optimized scaling factor using ABC and GA is also compared and presented in Table 12 in terms of NC and BER under zero attack. From Table 12, it can be studied that, for grayscale images, the performance of both schemes are at par for ABC and GA. However, for the color images, the proposed scheme has a higher NC value and lower BER value than [28], indicating that the proposed scheme has higher robustness for color images. From this discussion, it can be concluded that the proposed fitness function has improved scaling factor optimization compared to [28].

## 5. Conclusions

The proposed hybrid IWT-SVD DIW scheme is blind, secure and adaptive. Hence, it is suitable for robust transmission of digital images in public channels. Embedding the encrypted watermark in a randomly selected position of the cover image provides high watermark security, which makes the proposed scheme suitable for applications such as scientific documents and courtroom proof transmission, military applications, fingerprinting, telecoms, etc. Adaptive generation of ISF provides higher imperceptibility and robustness. Using NIO algorithms for ISF optimization further improves performance of watermarking characteristics. The proposed scheme is also free from FPE due to Pseudo random key and two-level adaptive embedding. The simulation results show that the proposed scheme provides higher watermarking characteristics and is able to sustain the majority of image processing attacks. Furthermore, comparative study with state-of-the-art schemes exhibits that the proposed scheme shows higher imperceptibility, robustness, security and embedding rate than state-of-the-art schemes. The fitness function proposed in the scheme is also more fitting. The proposed scheme can be used for copyright security, ownership verification, image authentication, telemedicine, military applications, transmission of scientific or courtroom documents, fingerprinting, image forensics, etc. Improving the embedding rate and robustness can be seen as future work. Hybrid optimization approaches for high exploitation and exploration for higher imperceptibility and robustness is also prospective research.

## Figures and Tables

**Table 1:** MSE, PSNR, SSIM, NC and BER (under zero attacks) for different test cover images using adaptive ISF.

Grayscale Image	MSE	PSNR (dB)	SSIM	NC	BER	Alpha
Lena	0.4315	51.67	0.9853	1	0	1.03417
Baboon	0.4395	51.58	0.9982	1	0	3.93945
Lighthouse	0.3755	52.41	0.9530	1	0	0.59863
Desert	0.3663	52.50	0.9657	1	0	0.68652
Grain	0.4573	51.52	0.9867	1	0	2.93261
MRI	0.3009	53.31	0.9035	1	0	0.57421
Avearage	0.3951	52.16	0.9654	1	0	
**Color** **Image**	**MSE**	**PSNR**	**SSIM**	**NC**	**BER**	**Apha**
Koala	0.1538	56.31	0.9991	0.9991	0.0022	2.33984
Penguins	0.1319	56.90	0.9990	0.9992	0.0015	0.48535
Tulips	0.9919	60.85	1		0.0001	0.41992
Tiffany	0.0865	58.76	0.9977	0.9901	0.0308	0.45898
Splash	0.1080	57.79	0.9976	0.9899	0.0229	1.07324
Skin	0.1336	56.75	0.9999	0.9992	0.0014	2.07812
Average	0.0112	57.89	0.9988	0.9949	0.0115	

**Table 2:** MSE, PSNR, SSIM, NC, BER (under zero attacks) and adaptive ISF for 50 test cover images taken from USC-SIPI.

Image	MSE	PSNR (dB)	SSIM	NC	BER	Alpha
Grass (1.1.01)	0.4129	52.03	0.9999	1	0	6.7734
Bark (1.1.02)	0.4362	51.74	0.9995	1	0	7.7979
Straw (1.1.03)	0.4399	51.71	0.9996	1	0	9.7490
Herringbone weave (1.1.04)	0.3934	52.18	0.9998	1	0	13.9102
Woolen (1.1.05)	0.4215	51.95	0.9992	1	0	6.5332
Pressed calf leather (1.1.06)	0.4194	51.89	0.9998	1	0	11.9004
Beach sand (1.1.07)	0.4356	51.79	0.9993	1	0	6.9023
Water (1.1.08)	0.3476	52.64	0.9969	1	0	2.9189
Wood grain (1.1.09)	0.4101	52.02	0.9983	1	0	2.7051
Raffia (1.1.10)	0.4186	51.95	0.9994	1	0	7.6084
Grass (1.2.03)	0.4472	52.78	0.9962	1	0	2.3681
Brick wall (1.2.12)	0.4522	52.54	0.9998	1	0	1.9532
Tile roof (1.4.05)	0.4119	51.98	0.9755	1	0	3.1567
Wood fence (1.4.06)	0.3810	52.32	0.9964	1	0	2.4517
Metal grates (1.4.07)	0.3897	52.24	0.9977	1	0	3.8203
.Female (4.1.01)	0.3085	53.19	0.9774	1	0	1.3779
Couple (4.1.02)	0.3041	53.28	0.9360	1	0	0.6797
Female (4.1.03)	0.2070	54.98	0.9424	1	0	0.4785
Female (4.1.04)	0.2912	53.52	0.9772	1	0	0.7607
. House (4.1.05)	0.2895	53.56	0.9681	1	0	1.2666
Tree (4.1.06)	0.3627	52.57	0.9731	1	0	1.6543
Jelly bean (4.1.07)	0.0899	58.59	0.9935	1	0	0.0332
Airplane (4.2.05)	0.4051	52.08	0.9697	1	0	0.9648
Sail boat (4.2.06)	0.4668	51.42	0.9909	1	0	1.6055
Peppers (4.2.07)	0.4632	51.49	0.9896	1	0	1.4355
Moon surface (5.1.09)	0.3321	52.92	0.9924	1	0	2.0449
Aerial (5.1.10)	0.4218	51.85	0.9961	1	0	2.6289
Airplane (5.1.11)	0.2134	54.81	0.9485	1	0	0.5557
Clock (5.1.12)	0.2541	54.07	0.9451	1	0	0.4590
Resolution chart (5.1.13)	0.1065	57.85	0.6537	1	0	0.0010
Chemical paint (5.1.14)	0.4021	52.07	0.9940	1	0	2.4258
Couple (5.2.08)	0.4415	51.68	0.9917	1	0	1.3770
Aerial (5.2.09)	0.4521	51.57	0.9936	1	0	1.8740
Stream and Bridge (5.2.10)	0.5907	50.43	0.9973	1	0	3.5762
Male (5.3.01)	0.4408	51.70	0.9909	1	0	1.5879
Airport (5.3.02)	0.4611	51.47	0.9925	1	0	1.6172
Truck (7.1.01)	0.4501	51.60	0.9925	1	0	1.6709
Airplane (7.1.02)	0.3204	53.02	0.9725	1	0	0.4961
Car (7.1.03)	0.4444	51.63	0.9948	1	0	2.1592
Car and APCs (7.1.04)	0.4155	51.89	0.9943	1	0	2.3555
Truck and APCs (7.1.06)	0.4430	51.66	0.9969	1	0	3.6641
Tank (7.1.07)	0.4351	51.79	0.9976	1	0	3.7002
APC (7.1.08)	0.4641	51.50	0.9891	1	0	1.2744
Tank (7.1.09)	0.4369	51.75	0.9972	1	0	2.9941
Tank and APCs (7.1.10)	0.4183	51,89	0.9958	1	0	2.6553
Airplane (7.2 01)	0.4031	52.03	0.9767	1	0	0.8965
Fishing boat	0.4486	51.57	0.9930	1	0	3.8765
Level step wedge	0.3216	52.87	0.9973	1	0	2.3144
House	0.4143	51.94	0.9677	1	0	1.9874
Pixel ruler	0.0924	58.47	0.8453	1	0	1.7432
Average	0.3813	51.55	0.9767	1	0	

**Table 3:** PSNR, SSIM using optimized ISF with GA, ABC, FO.

Grayscale Images	With GA			With ABC			With FO		
	PSNR (dB)	SSIM	Alpha	PSNR (dB)	SSIM	Alpha	PSNR (dB)	SSIM	Alpha
Lena	53.14	0.9894	9.18692	52.32	0.9871	4.2647	52.29	0.9869	4.47517
Mandrill	51.96	0.9984	8.84809	51.60	0.9983	4.2157	51.60	0.9983	4.04169
Lighthouse	53.44	0.9597	9.34731	52.92	0.9570	4.1358	52.79	0.9560	3.63865
Desert	53.34	0.9713	6.06826	53.09	0.9707	4.2014	53.04	0.9706	3.82451
Grain	51.52	0.9862	6.18985	51.54	0.9866	4.0224	51.57	0.9845	5.04537
MRI	54.27	0.9062	5.69925	54.07	0.9059	3.8877	53.88	0.9055	2.70833
Average	52.95	0.9685		52.59	0.9676		52.52	0.9669	
**Color** **Images**	**PSNR**	**SSIM**	**Apha**	**PSNR**	**SSIM**	**Apha**	**PSNR**	**SSIM**	**Apha**
Koala	57.11	0.9993	9.62690	56.56	0.9991	4.7327	56.48	0.9991	4.1267
Penguins	57.66	0.9990	9.88692	57.21	0.9990	4.7606	57.17	0.9990	3.9985
Tulips	61.50	1	6.67544	61.37	1	5.1535	61.35	1	4.9423
Tiffany	57.46	0.9978	9.80044	59.22	0.9978	4.7247	59.32	0.9972	4.2167
Splash	58.14	0.9976	5.32084	58.12	0.9977	6.5745	58.03	0.9976	5.9291
Skin	58.68	0.9999	9.26637	57.81	0.9999	4.8773	57.60	0.9999	
Average	58.42	0.9989		58.38	0.9989		58.32	0.9988	

**Table 4:** NC, BER under zero attack using optimized ISF with GA, ABC and FO.

Greyscale Imagee	With GA			With ABC			With FO		
	**NC**	**BER**	**Apha**	**NC**	**BER**	**Apha**	**NC**	**BER**	**Apha**
Lena	1	0	9.18692	1	0	4.2647	1	0	4.47517
Baboon	1	0	8.84809	1	0.0004	4.2157	1	0	4.04169
Lighthouse	1	0	9.34731	1	0.0002	4.1358	1	0	3.63865
Desert	1	0	6.06826	1	0.0002	4.2014	1	0	3.82451
Grain	1	0	6.18985	1	0	4.0224	1	0	5.04537
MRI	1	0	5.69925	1	0	3.8877	1	0	2.70833
Average	1	0		1	0.0001		1	0	
**Color** **Images**	**NC**	**BER**	**Apha**	**NC**	**BER**	**Apha**	**NC**	**BER**	**Apha**
Koala	0.9999	0	9.62690	0.9996	0.0004	4.7327	0.9996	0	4.1267
Penguins	1	0.0004	9.88692	0.9997	0.0004	4.7606	0.9996	0.0004	3.9985
Tulips	1	0.0002	6.67544	1	0	5.1535	1	0	4.9423
Tiffany	0.9997	0.0002	9.80044	0.9990	0.0004	4.7247	0.9993	0	4.2167
Splash	0.9995	0	5.32084	0.9997	0	6.5745	0.9990	0	5.9291
Skin	0.9999	0	9.26637	0.9996	0	4.8773	0.9996	0	4.5178
Average	0.9998	0.0001		0.9996	0.0002		0.9991	0	

**Table 5:** NC, BER with adaptive ISF (alpha), GA, ABC and FO under common attacks for Lena Image.

Attacks	With ISF (Alpha = 1.0341)		With GA (Alpha = 9.18619)		With ABC (Alpha = 4.2647)		With FO (Alpha = 4.4752)	
	NC	BER	NC	BER	NC	BER	NC	BER
Original	1	0.0004	1	0	1	0	1	0
Salt and Pepper (0.002)	0.6954	0.5710	**0.8592**	0.5610	0.7701	0.5706	0.6859	0.5708
Gaussian Noise (0.0002)	0.9472	0.5710	**0.9671**	0.5551	0.9601	0.5640	0.9538	0.5701
Speckle Noise (0.0002)	0.9426	0.5710	**0.9676**	05624	0.9602	0.5668	0.9539	0.5702
Poisson Noise	0.9460	0.5710	**0.9679**	0.5590	0.9599	0.5623	0.9556	0.5689
Cropping (25 % )	0.9378	0.5102	**0.9566**	0.4956	0.9500	0.5083	0.9439	0.4934
Rotate_ 45 (clockwise)	0.9703	0.0332	**0.9958**	0.0012	0.9897	0.0146	0.9909	0.0104
Rotate _ 10 (clockwise)	0.9993	0.0031	**0.9997**	0.0004	0.9994	0.0007	0.9994	0.0004
Translate (24.3, 10.1)	0.9905	0.0078	**0.9990**	0.0007	0.9983	0.0012	0.9982	0.0012
Resize (256)	0.9410	0.0078	**0.9725**	0.0007	0.9594	0.0012	0.9542	0.0010
Resize (320)	0.9835	0.0400	**0.9950**	0.0048	0.9905	0.0266	0.9910	0.0263
Jpeg Compression (60%)	0.9880	0.0183	**0.9989**	0.0004	0.9961	0.0065	0.9957	0.0041
Sharpening	0.9973	0.1269	**0.9991**	0.1054	0.9987	0.1293	0.9990	0.1396
Gaussian Filter (3 by 3)	1	0.0004	1	0	1	0.0004	1	0
Median Filter (3 by 3)	0.9952	0.0017	**0.9976**	0.0007	0.9973	0.0004	0.9970	0.0003
Average Filter (3 by 3)	0.9608	0.3940	**0.9853**	0.4416	0.9764	0.4118	0.9725	0.3950
Average Filter (5 by 5)	0.8555	0.4206	0.9119	0.3798	0.9027	0.3999	**0.9922**	0.0009
Weiner Filter (3 by 3)	0.9745	0.0146	**0.9986**	0.0004	0.9966	0.0034	0.9783	0.0144
Butter worth Filter (Threshold = 20, Grade = 1)	0.9602	0.5710	**0.9895**	0.4523	0.9854	0.4610	0.9876	0.4598
Gamma Correctoin (0.25)	0.9983	0.0021	**0.9993**	0.0004	0.9990	0.0012	0.9992	0.0009
Gamma Correction (0.3)	0.9983	0.0021	**0.9994**	0.0008	0.9991	0.0008	0.9990	0.0012
Shear (x = 1, y = 0.2)	0.9972	0.0009	**0.9983**	0.0004	0.9981	0.0004	0.9980	0.0004

**Table 6:** CC between original, encrypted images and original, decrypted images.

Test Images (Binary)	Correlation of Original and Encrypted Images			Correlation of Original and Decrypted Images		
	Horizontal	Vertical	Diagonal	Horizontal	Vertical	Diagonal
Cameraman	0.1185	0.1263	0.0721	1	1	1
Trishool	0.1238	0.1628	0.1828	1	1	1
Koala	0.1472	0.1577	0.0165	1	1	1
Lena	0.1294	0.1376	0.0938	1	1	1
Penguins	0.1435	0.1237	0.1171	1	1	1

**Table 7:** CC between two encrypted images with one bit differ in Random key.

Original Images	Correlation between Two Encrypted Images		
	Horizontal	Vertical	Diagonal
Cameraman	−0.0281	−0.0173	−0.0611
Trishool	−0.0248	−0.0167	−0.0231
Koala	−0.0173	−0.0104	0.0057
Lena	−0.0086	0.0303	0.0017
Penguins	−0.0253	−0.0778	−0.0237

**Table 8:** Computational time of major algorithmic steps for the cover image of size M×N.

Operations	Computational Cost
1-level 2D IWT transform	O(2MN)
1-level 2D inverse IWT transform	O(2MN)
SVD decomposition	$O(2MN^{2}+2N^{3})$
SVD re-composition	O(2min[M,N]MN)
ISF Optimization	O(MN)
Adaptive block selection	O(MN)
Determination of adaptive embedding position using BBS	O(log(logm))

**Table 9:** Embedding and extraction time (seconds) of cameraman watermark image with different test cover images.

Grayscale Image	Embedding Time (s)	Extraction Time (s)	Color Image	Embedding Time (s)	Extraction Time (s)
Lena	1.919406	1.056093	Koala	1.378239	0.987560
Baboon	1.465614	0.915152	Penguins	1.686724	0.977352
Lighthouse	1.553255	1.025732	Tulips	1.433371	0.831694
Desert	1.324148	0.985283	Tiffany	1.277285	0.900063
Grain	1.264661	0.973439	Splash	1.244865	0.907733
MRI	1.470474	0.715602	Skin	1.273823	0.616617
Average	1.526388	0.995216	Average	1.382384	0.870169

**Table 10:** Watermarking parameters of proposed scheme and state-of-the-art schemes [18,23,27,31,32].

Parameters	Ansari and Pant. [18]	Moeinnaddini. [31]	Singh and Bhatnagar. [32]	Sharma and Mir. [27]	Zainol et al. [23]	Proposed
Scheme	Non-blind	Blind	Blind	Blind	Blind	Blind
Embedding domain	DWT + SVD	Hadmard	LWT + d-sequence	DCT	IWT + SVD	IWT + SVD
Cover image size	512 by 512	512 by 512	512 by 512	512 by 512	512 by 512	512 by 512
Watermark size	64 by 64	64 by 64	16 by 16	64 by 64	256 by 256	64 by 64
Security	Yes	Yes	Yes	No	Yes	Yes
Encryption approach	Arnold	No	Arnold	No	Chaotic map	Pseudo random key
Optimization algorithm	ABC	DDFA	No	ACO	No	GA, ABC, FA
Handling FPE	No	Yes	Yes	Yes	Yes	Yes
Embedding position	Principal component	Coefficients adjustments	Sub bands	DC component	Principal component	Principal component
Embedding type	Static	Dynamic	Static	Dynamic	Static	Dynamic
Embedding rate	0.015625	0.015625	0.00097	0.015625	0.25	0.015625

**Table 11:** PSNR (dB) and SSIM for proposed and [28] using ABC and GA.

	ABC Proposed		ABC [28]		GA Proposed		GA [28]	
	**PSNR**	**SSIM**	**PSNR**	**SSIM**	**PSNR**	**SSIM**	**PSNR**	**SSIM**
Grayscale Image								
Lena	52.32	0.9971	47.45	0.9964	53.14	0.9894	50.81	0.9825
Baboon	51.60	0.9983	50.86	0.9961	51.96	0.9984	51.02	0.9931
Lighthouse	52.92	0.9570	52.37	0.9534	53.44	0.9597	51.89	0.9532
Desert	53.09	0.9707	52.61	0.9682	53.34	0.9713	51.95	0.9647
Grain	51.54	0.9866	51.04	0.9836	51.52	0.9862	50.73	0.9806
MRI	54.07	0.9059	53.06	0.9026	54.27	0.9062	53.01	0.9029
Color Images								
Koala	56.56	0.9991	49.11	0.9925	57.11	0.9993	56.02	0.999
Penguins	57.21	0.999	51.51	0.9998	57.66	0.999	56.02	0.9982
Tulips	61.37	1	48.18	0.9997	61.50	1	59.98	0.9999
Tiffany	59.22	0.9978	58.09	0.9977	57.46	0.9978	58.04	0.9977
Splash	58.12	0.9977	56.08	0.9976	58.14	0.9976	56.04	0.9976
Skin	57.81	0.9999	56.83	0.9998	58.68	0.9999	55.83	0.9998

**Table 12:** NC and BER for the proposed scheme and [28] using ABC and GA under zero attack.

	ABC Proposed		ABC [28]		GA Proposed		GA [28]	
	NC	BER	NC	BER	NC	BER	NC	BER
Grayscale Image								
Lena	1	0	1	0.0004	1	0	1	0
Baboon	1	0.0004	1	0.0004	1	0	1	0
Lighthouse	1	0.0002	1	0.0002	1	0	1	0
Desert	1	0.0002	1	0	1	0	1	0
Grain	1	0	1	0	1	0	1	0
MRI	1	0	1	0	1	0	1	0
Color Images								
Koala	0.9996	0.0004	0.9991	0.0019	0.9999	0	0.9977	0.0019
Penguins	0.9997	0.0004	0.9980	0.0017	1	0.0004	0.9991	0.0008
Tulips	1	0	0.9981	0.0009	1	0.0002	0.9961	0.0042
Tiffany	0.9998	0.0004	0.9903	0.0096	0.9997	0.0002	0.991	0.0351
Splash	0.9997	0	0.9896	0.0094	0.9995	0	0.9903	0.0093
Skin	0.9996	0	0.9950	0.0032	0.9999	0	0.9963	0.0059

## Data Availability

Not applicable.
